# Isolation and Characterization of the Novel Botulinum Neurotoxin A Subtype 6

**DOI:** 10.1128/mSphere.00466-18

**Published:** 2018-10-24

**Authors:** Molly S. Moritz, William H. Tepp, Marite Bradshaw, Eric A. Johnson, Sabine Pellett

**Affiliations:** a Department of Bacteriology, University of Wisconsin, Madison, Wisconsin, USA; University of Maryland Medical Center

**Keywords:** BoNT, BoNT/A6, botulinum neurotoxin, cell entry, duration, potency, subtype

## Abstract

Botulinum neurotoxins (BoNTs), the most potent toxins known to humans and the causative agent of botulism, exert their effect by entering motor neurons and cleaving and inactivating SNARE proteins, which are essential for neurotransmitter release. BoNTs are proven, valuable pharmaceuticals used to treat more than 200 neuronal disorders. BoNTs comprise 7 serotypes and more than 40 isoforms (subtypes). BoNT/A1 is the only A-subtype used clinically due to its high potency and long duration of action. While other BoNT/A subtypes have been purified and described, only BoNT/A2 is being investigated as an alternative to BoNT/A1. Here we describe subtype BoNT/A6 with improved pharmacological properties compared to BoNT/A1. It was isolated from Clostridium botulinum CDC41370, which produces both BoNT/B2 and BoNT/A6. The gene encoding BoNT/B2 was genetically inactivated, and A6 was isolated to greater than 95% purity. A6 was highly potent in cultured primary rodent neuronal cultures and in human induced pluripotent stem cell-derived neurons, requiring 20-fold less toxin to cause 50% SNAP-25 cleavage than A1. Second, A6 entered hiPSCs faster and more efficiently than A1 and yet had a long duration of action similar to BoNT/A1. Third, BoNT/A6 had similar LD_50_ as BoNT/A1 after intraperitoneal injection in mice; however, local intramuscular injection resulted in less systemic toxicity than BoNT/A1 and a higher (i.m.) LD_50_, indicating its potential as a safer pharmaceutical. These data suggest novel characteristics of BoNT/A6 and its potential as an improved pharmaceutical due to more efficient neuronal cell entry, greater ability to remain localized at the injection site, and a long duration.

**IMPORTANCE** Botulinum neurotoxins (BoNTs) have proved to be an effective treatment for a large number of neuropathic conditions. BoNTs comprise a large family of zinc metalloproteases, but BoNT/A1 is used nearly exclusively for pharmaceutical purposes. The genetic inactivation of a second BoNT gene in the native strain enabled expression and isolation of a single BoNT/A6 from cultures. Its characterization indicated that BoNT/A subtype A6 has a long duration of action comparable to A1, while it enters neurons faster and more efficiently and remains more localized after intramuscular injection. These characteristics of BoNT/A6 are of interest for potential use of BoNT/A6 as a novel BoNT-based therapeutic that is effective and has a fast onset, an improved safety profile, and a long duration of action. Use of BoNT/A6 as a pharmaceutical also has the potential to reveal novel treatment motifs compared to currently used treatments.

## Abstract (precis)

Botulinum neurotoxins (BoNTs) have proved to be an effective treatment for a large number of neuropathic conditions. BoNTs comprise a large family of zinc metalloproteases, but BoNT/A1 is used nearly exclusively for pharmaceutical purposes. The genetic inactivation of a second BoNT gene in the native strain enabled expression and isolation of a single BoNT/A6 from cultures. Its characterization indicated that BoNT/A subtype A6 has a long duration of action comparable to A1, while it enters neurons faster and more efficiently and remains more localized after intramuscular injection. These characteristics of BoNT/A6 are of interest for potential use of BoNT/A6 as a novel BoNT-based therapeutic that is effective and has a fast onset, an improved safety profile, and a long duration of action. Use of BoNT/A6 as a pharmaceutical also has the potential to reveal novel treatment motifs compared to currently used treatments.

## INTRODUCTION

Botulinum neurotoxins (BoNTs) are the most poisonous toxins known to humans. BoNTs are designated Tier 1 Select Agents, and their production, isolation, and characterization require CDC-approved laboratory facilities. They are produced by Clostridium botulinum and select strains of Clostridium butyricum, Clostridium sporogenes, Clostridium argentinense, and Clostridium baratii (1, 2). They comprise seven immunologically distinct BoNTs (A to G) and at least 40 isoforms (subtypes). BoNTs are synthesized as 150-kDa dichain proteins comprised of a 100-kDa heavy chain (HC) and a 50-kDa light chain (LC) linked by a disulfide bond. The HC has a C-terminal domain (H_C_), which recognizes and binds to gangliosides and protein receptors on neuronal cells, and an N-terminal domain (H_N_), which functions in translocation of the LC into the cell cytosol (3–5). Once inside the cell, the LC specifically cleaves a soluble *N*-ethylmaleimide-sensitive factor attachment protein receptor (SNARE) protein, thereby inactivating neurotransmitter release (6, 7). This leads to the characteristic flaccid paralysis of botulism, which can last several days to months depending on BoNT sero- and subtype and the dose. The long duration of action is due to the persistence and continued catalytic activity of the LC inside neurons (8). While BoNTs have the potential to cause serious illness, they are used in the effective treatment of a myriad of neuronal disorders, providing therapy to numerous diseases, many of which were previously untreatable (9).

BoNTs are divided into seven serotypes (A to G) which are further categorized into subtypes based on variations in amino acid sequences (1, 10–13). Despite the multitude of BoNT subtypes, only two are being employed as pharmaceuticals, namely, BoNT/A1 and to a much lesser extent BoNT/B1. This is in part due to the unavailability of the purified BoNT subtypes, and a corresponding dearth of knowledge of pharmaceutical properties of most BoNT subtypes. Only a few BoNT subtypes have been isolated and characterized for their biochemical, cellular, and *in vivo* properties. Most research has focused on BoNT/A subtypes. *In vitro* and *in vivo* investigations of BoNT/A subtypes 1 to 5 isolated in our and other laboratories have revealed unique properties of some subtypes, including potency, cell entry kinetics, duration of action, and cellular mechanisms governing these properties (13–16). In an earlier study in our laboratory, BoNT/A2 was shown to enter neuronal cells faster and more efficiently than BoNT/A1 (15, 16). *In vivo* studies in mice comparing purified BoNT/A1 and A2 indicated similar potency and duration of action and a slightly faster onset of local paralysis by A2 (17). A further study compared a pharmaceutical preparation of purified BoNT/A2 with pharmaceutically formulated BoNT/A1 complex in mice after local intramuscular injection. This study indicated that the A2 product was more potent than the A1 product for causing local paralysis and remained more localized within the injection site, suggesting a lower risk for detrimental side effects (18–20). As a result of these data, BoNT/A2 was suggested as another subtype of choice for therapeutic treatment (18, 19) and is currently in clinical trials in Japan (20).

In addition to the biochemical and functional characterizations of BoNT/A1 to -5, BoNT/A subtypes A7 and A8 have recently been partially characterized *in vitro* (21, 22). However, for subtype BoNT/A6 little is known of its composition and functional properties, largely because BoNT/A6 is produced in a strain that produced two BoNTs, B2 and A6 (23, 24). This complicates its isolation and characterization. BoNT/A6 differs from other BoNT/A subtypes in its amino acid sequence by 4% to 14%, with the greatest similarity to A1 (95.7%) and A5 (95.9%) and the greatest dissimilarity to A3 (86%) (23, 25). BoNT/A6 was first described in 2009 as a unique BoNT/A subtype, possibly derived from a recombination event of BoNT/A1 and A2 (23). The LC sequence of BoNT/A6 differs from that of A1 by only 1 amino acid residue outside any exosite region (T414A), while the translocation and receptor binding domains differ by 4.5 and 9.5%, respectively. The majority of the differences of the A6 translocation domain from A1 are also present in A2, but the receptor binding domain of A6 has several unique amino acid variations (see Fig. S1 in the supplemental material).

10.1128/mSphere.00466-18.1 FIG S1 Protein sequence alignment of BoNTs A1, A2, and A6. Clustal Omega was used to compare the sequences of BoNT/A1 (CAL82360.1), /A2 (CAA51824.1), and /A6 (ACW83608.1). The protein sequences were entered into the multiple sequence alignment program, and the output format was set to ClustalW with character counts. Variations among the sequences are indicated. Download FIG S1, PDF file, 0.03 MB. Copyright © 2018 Moritz et al. 2018 Moritz et al. This content is distributed under the terms of the Creative Commons Attribution 4.0 International license.

This study describes the isolation of BoNT/A6 and its characterization both *in vitro* and *in vivo*. The results show higher potency and faster cell entry kinetics of BoNT/A6 in cultured neuronal cell models compared to other BoNT/A subtypes, with similarities to A2 (16). Mouse studies indicate a faster onset and a long duration of action after local intramuscular injection comparable to BoNT/A1. These results indicate novel fundamental pharmacological properties of BoNT/A6 and its potential as an improved pharmaceutical.

## RESULTS

### BoNT/A6 was independently expressed and purified from its native strain.

To facilitate purification and characterization of BoNT/A6 from its native dual-toxin-producing C. botulinum strain CDC41370, production of BoNT/B2 in this strain was genetically eliminated by inactivation of the B2 toxin gene using ClosTron mutagenesis system as previously described for C. botulinum strain 69016 producing BoNT/B2 and BoNT/FA (see Fig. S2A in the supplemental material) (24). Inactivation of the BoNT/B2 gene was confirmed by PCR (Fig. S2B), sequencing, and Southern hybridization (Fig. S2C to E). The mutant strain contained one copy of the intron element inserted into the targeted position in the *bont/B2*. The mutant strain was designated CDC41370/B2^tox-^. Western blot analysis of the wild-type and the mutant strain cultures with anti-BoNT/A1 antibodies revealed expression of the 150-kDa BoNT/A6, which was proteolytically activated to a 50-kDa light chain (LC) and a 100-kDa heavy chain (HC) in the culture. Western blotting using anti-BoNT/B1 antibodies, which strongly recognize BoNT/B2, indicated no expression of BoNT/B (Fig. S3). These data confirmed that the CDC41370/B2^tox-^ strain produced BoNT/A6 but not BoNT/B2 at levels detectable by immunoblotting.

10.1128/mSphere.00466-18.2 FIG S2 Mutant screening by PCR and Southern hybridization. (A) Schematic presentation of the wild-type (top) and the mutated (bottom) botulinum neurotoxin B2 gene. The group II intron is shown as a black wide arrow inserted on the sense strand of the toxin gene (gray arrow) between nucleotides 381 and 382 as indicated by vertical arrows. The white arrow inside the intron element in opposite orientation to the intron and toxin gene is a retrotransposition-activated erythromycin (RAM-Erm) resistance gene. The locations of the PCR primers F and R are shown with horizontal arrows on either side of the intron insertion site. The expected size of the PCR products for the wild-type strain is 238 bp and 2,019 bp for the inactivated BoNT/B2 gene. (B) PCR products of eight putative mutant clones (lanes 1 to 8) and a wild-type (WT) strain. M, GeneRuler 1-kb DNA ladder (Thermo Scientific). (C to E) Southern hybridization: (C) with the intron probe (erythromycin gene) and (D) BoNT/B2probe. (E) Ethidium bromide-stained 1% agarose gel of genomic DNA digested with restriction enzyme HindIII. Lanes 1 and 2, two individual putative BoNT/B2 mutant clones; lane 3, wild-type CDC41370 strain; lane 4, DNA marker, lambda DNA HindIII digest (NEB, Ipswich, MA); lane 5, DNA marker, GeneRuler 1-kb DNA ladder (Thermo Scientific); the size of the DNA markers is indicated on the right side of the gel. In the wild-type strain CDC41370, a 946-bp HindIII DNA fragment is expected to hybridize with a BoNT/B2 gene probe (D) and with a 2,727-bp fragment in the mutant strain with an intron integrated into a BoNT/B2 gene between nucleotides 381 and 382. The erythromycin gene probe (C) hybridizes with the same 2,727-bp fragment in the mutant clones, and no hybridization signal is observed in the wild-type strain. Download FIG S2, TIF file, 0.8 MB. Copyright © 2018 Moritz et al. 2018 Moritz et al. This content is distributed under the terms of the Creative Commons Attribution 4.0 International license.

10.1128/mSphere.00466-18.3 FIG S3 Western analysis of neurotoxin expression in C. botulinum wild-type strain CDC41370 and BoNT/B2 gene mutant clones. (Left panel) Coomassie blue-stained SDS-PAGE gel. (Middle panel) Western blotting using antibodies raised against serotype A1 botulinum neurotoxin. (Right panel) Western blotting using antibodies raised against serotype B1 botulinum neurotoxin. Lanes M1 and M2, two individual mutant clones; WT, wild-type strain CDC41370. Purified botulinum neurotoxins BoNT/A1 and BoNT/B1 were used as standards. Abbreviations: R, reduced; NR, nonreduced; BoNT/SC, single-chain botulinum neurotoxin; BoNT/LC, botulinum neurotoxin light chain; BoNT/HC, botulinum neurotoxin heavy chain. Only reduced wild-type and the mutant clone total culture samples were analyzed. The wild type and both mutant clones express BoNT/A6; however, only the wild-type strain expresses BoNT/B2. This indicated the mutant clones no longer express the second toxin, BoNT/B2. Download FIG S3, TIF file, 0.9 MB. Copyright © 2018 Moritz et al. 2018 Moritz et al. This content is distributed under the terms of the Creative Commons Attribution 4.0 International license.

**FIG 1:**
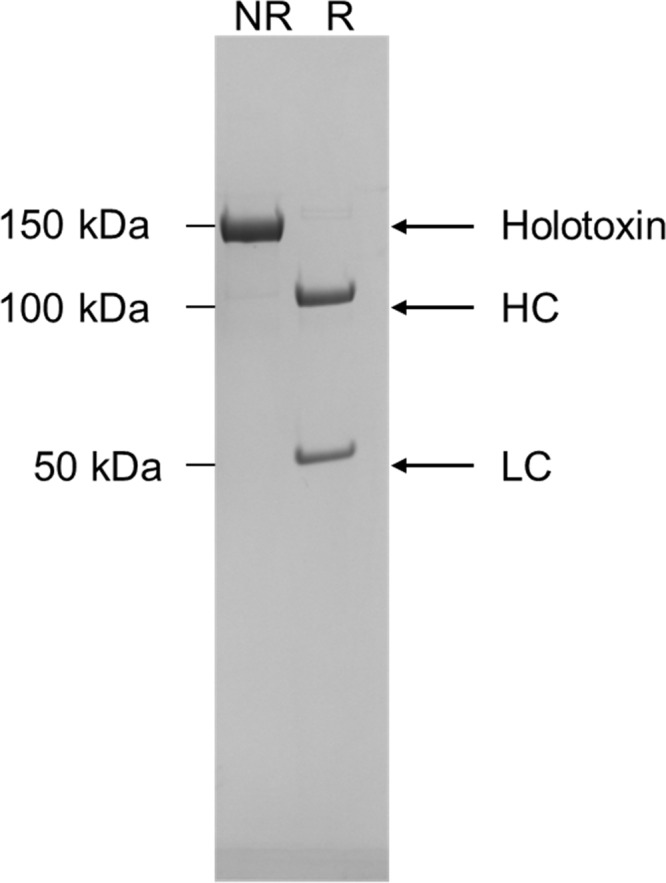
Isolation of BoNT/A6. The Clostridium botulinum strain CDC41370 was genetically modified to no longer produce BoNT/B2 as described in Materials and Methods. The purity of BoNT/A6 was verified by this SDS-PAGE gel showing the nonreduced (NR) holotoxin and reduced (R) heavy chain (HC) and light chain (LC).

Strain CDC41370/B2^tox-^ was used to produce purified BoNT/A6 using previously described methods (15, 26, 27). A 10-liter starting culture resulted in a final yield of 9.5 mg of BoNT/A6 as determined by spectroscopy and SDS-PAGE gel analysis (Fig. 1). This is a similar yield from purification of an A1 Hall strain culture, which generally yields ∼10 to 15 mg of purified BoNT/A1. Densitometry analysis of an SDS-PAGE gel indicated that the isolated BoNT/A6 was ∼95% pure and was fully nicked to its dichain form (Fig. 1). A standard mouse bioassay was used to determine the specific activity of BoNT/A6. Deaths of mice were recorded through 4 days postinjection, and any mice that survived 48 h without botulism symptoms and appeared healthy were euthanized after 4 days. The LD_50_ was estimated to be 5.3 pg. This value is defined as 1 unit for this specific stock of BoNT/A6.

### The catalytic LC of BoNT/A6 has similar activity as BoNT/A1 LC.

**FIG 2:**
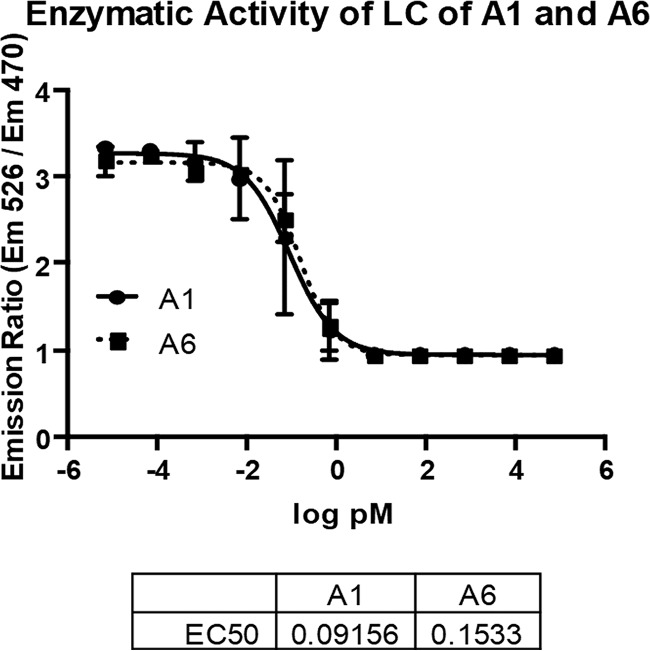
LC activity of BoNT/A1 and /A6 after 2 h. Serial dilutions of BoNT/A1 and /A6 were analyzed for light chain activity using a FRET endopeptidase assay (BoTest, Biosentinel). Proteolytic activity of BoNT/A in real time was detected using 3 independent experiments, and averages and standard deviations from the three independent assays are shown. The BoTest uses a truncated SNAP-25 reporter construct of amino acids 141 to 206 flanked by a cyan florescent protein (CFP) and a yellow fluorescent protein (YFP), which leads to fluorescence emissions reflecting cleavage of SNAP-25.

Enzymatic activities of the light chains of BoNT/A1 and /A6 were examined by FRET assay using a SNAP-25 fragment (aa141 to aa206) surrounded by a cyan fluorescent protein (CFP) and a yellow fluorescent protein (YFP) (BoTest; Biosentinel). As expected based on the almost 100% amino acid identity of these two LCs, there was no significant difference between the activities of LC/A1 and LC/A6 after 2 h (Fig. 2). The 50% effective concentration was determined to be 0.09 pM (95% confidence interval: 0.07 to 0.12) for A1 and 0.15 pM (95% confidence interval: 0.12 to 0.2) for A6 by analyzing the averages and standard deviations of each toxin concentration from the three independent assays. Similar data were observed with longer (up to 21 h) incubations (data not shown).

### BoNT/A6 has a high potency in neuronal cells.

**FIG 3:**
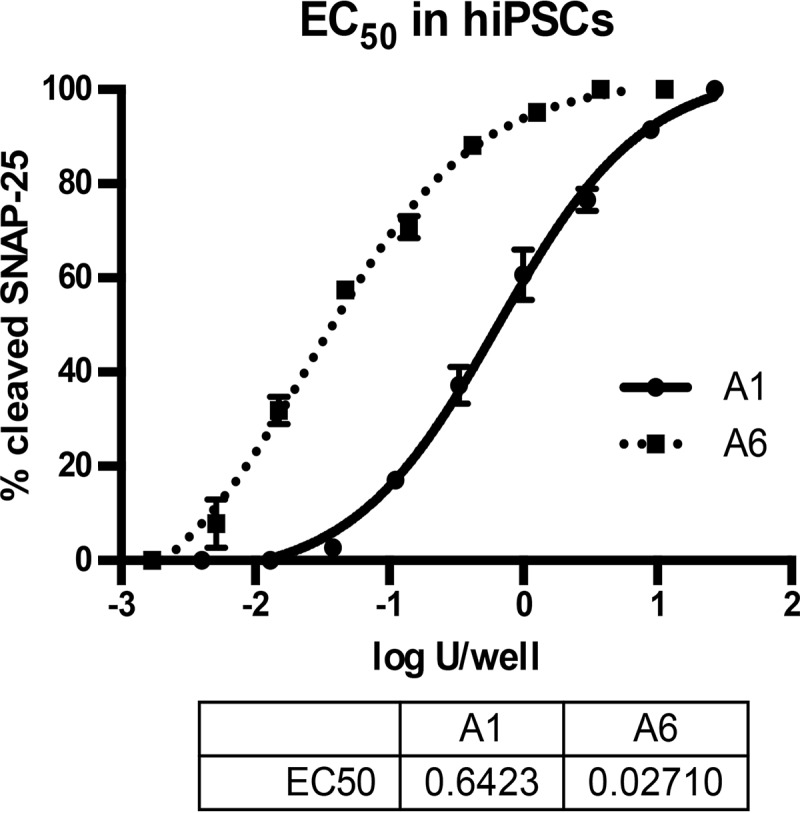
SNAP-25 cleavage of BoNT/A1 and /A6 in hiPSCs. hiPSCs were exposed to serial dilutions of either A1 or A6 for 48 h. Cell lysates were analyzed for cleaved and uncleaved SNAP-25 by Western blotting and densitometry. Averages and standard deviations of triplicate samples are shown. The EC_50_s were determined in PRISM6 software using a nonlinear regression (four parameters).

Previous studies have described the potency of BoNT/A subtypes 1 through 5 in various cell models (15, 16). Exposure of cultured mouse and rat primary spinal cord cells (MSC and RSC cells) to serial dilutions of BoNT/A6 resulted in a SNAP-25 cleavage pattern similar to that previously observed for BoNT/A2, with 50% SNAP-25 cleavage occurring at ∼0.03 U/50 µl/well (27 fM) after 48 h of toxin exposure (Fig. S4). The EC_50_ of BoNT/A6 was also determined in human iPSC-derived neurons. As with other BoNT/A subtypes, exposure of hiPSC neurons resulted in a steep dose-response curve extending from 0% to 100% cleavage within less than 4 logs for cells exposed to toxin for 48 h. The EC_50_ was calculated to be ∼0.03 U/50 µl/well (Fig. 3), which is about 20-fold more sensitive than was seen in a parallel experiment for BoNT/A1 (0.6 U/50 µl/well). These data indicate that BoNT/A6 is significantly more potent in cultured neurons than BoNT/A1, especially in human neurons.

10.1128/mSphere.00466-18.4 FIG S4 EC_50_ of BoNT/A6 in primary spinal cord cells. Primary mouse and rat spinal cord cells were exposed to serial dilutions of BoNT/A6 in culture medium for 48 hours. Cell lysates were analyzed for cleaved and uncleaved SNAP-25. The EC_50_ of BoNT/A6 was the same in both MSCs and RSCs, with ∼0.03 units required for cleavage of 50% of the SNAP-25 within 48 hours of toxin exposure. The EC_50_ values seen in these cell cultures were similar to what was seen for BoNT/A6 in hiPSCs. Download FIG S4, TIF file, 0.2 MB. Copyright © 2018 Moritz et al. 2018 Moritz et al. This content is distributed under the terms of the Creative Commons Attribution 4.0 International license.

### BoNT/A6 enters cells more efficiently than other BoNT/A subtypes.

**FIG 4:**
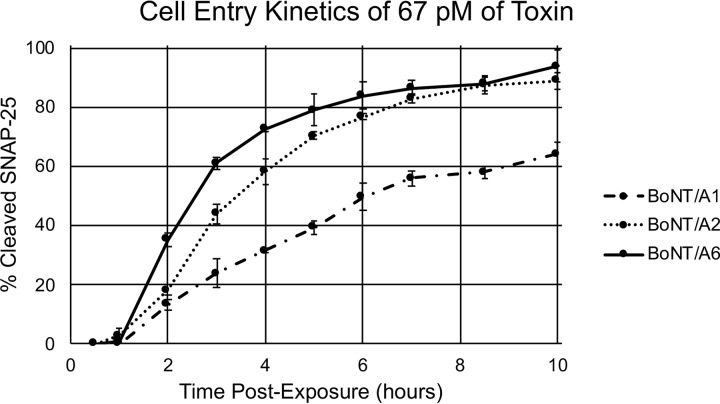
Cell entry kinetics of BoNT/A6 compared to /A1 and /A2. hiPSC-derived neurons were exposed to 70 pM BoNT/A1, /A2, or /A6 for up to 10 h, and cell lysates were prepared and analyzed for SNAP-25 cleavage at the indicated time points.

To examine the cell entry kinetics of BoNT/A6 compared to subtypes BoNT/A1 and /A2, hiPSC-derived neurons were exposed to 67 pM of each of the toxins (Fig. 4). Cells were harvested at the indicated time points through 10 h postexposure. Lysates were examined for the amount of cleaved and uncleaved SNAP-25 by Western blotting and densitometry. The onset of SNAP-25 cleavage occurred more rapidly in cells exposed to BoNT/A6 and BoNT/A2 than BoNT/A1 (Fig. 4). Cells exposed to BoNT/A6 also experienced an average of 94% ± 5.3% SNAP-25 cleavage at the final 10-hour time point, while cells exposed to BoNT/A1 and /A2 reached approximately 64% ± 4% and 84% ± 1.2% cleavage, respectively. While equimolar amounts of toxin were used in this assay, the three toxins had similar specific activities (5.6 pg/LD_50_ for A1, 4.9 pg/LD_50_ for A2, and 5.3 pg/LD_50_ for A6); thus, the results also compared similar numbers of biologically active units. This indicates that BoNT/A6 has an earlier onset of activity in cultured neurons compared to BoNT/A1 and similar to or even faster than BoNT/A2. Since catalytic activities of the BoNT/A6 and A1 LCs are similar, the earlier onset of activity in cultured neurons is likely due to faster or more efficient cell entry.

### BoNT/A6 has a similarly long persistence of activity in cultured rat and human neurons as BoNT/A1.

**FIG 5:**
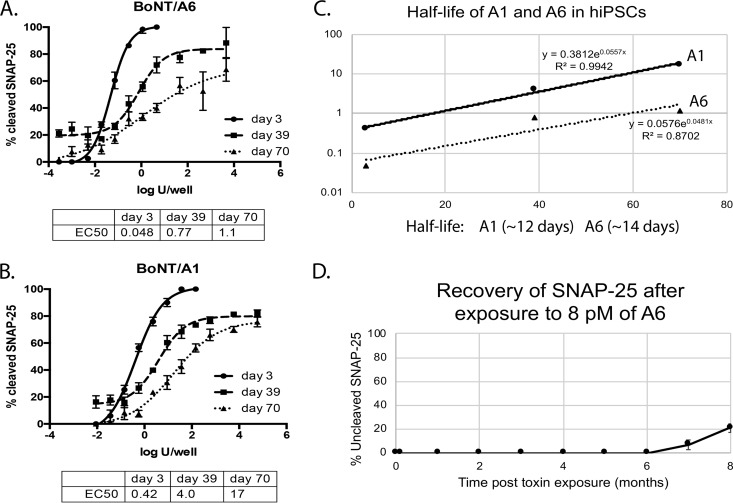
EC_50_ and duration of action of BoNT/A6 and /A1 in neurons. Human iPSC-derived neurons were exposed to serial dilutions of BoNT/A6 (A) or /A1 (B) for 72 h, followed by BoNT removal. Cells were incubated further in toxin-free medium and harvested on days 3, 39, and 70 after exposure. Graphs generated in PRISM6 software depicting the average and standard deviation of triplicate samples are shown (A and B). The EC_50_ values were determined in PRISM6 using a nonlinear regression (four parameters). The half-lives of BoNT/A1 and /A6 in hiPSCs were estimated by plotting the EC_50_ values versus time and using the formula *t*_1/2_ = ln(2)/slope of regression line (C). The half-lives were similar, being ∼12 and ∼14 days for A1 and A6, respectively. The duration of action was also evaluated in RSCs (D). RSCs were exposed to 8 pM BoNT/A6 for 3 days, followed by complete toxin removal. Cells were incubated for a longer duration in toxin-free medium, and cell lysates were prepared and analyzed for cleaved/uncleaved SNAP-25 by Western blotting and densitometry monthly until 8 months post-toxin exposure. Averages and standard deviations of quadruplicate samples are shown.

A previous report has shown that LC activities of BoNT/A1, /A2, /A4, and /A5 persist in cultured primary rat spinal cord neurons for over 10 months, and that the recovery rate for uncleaved SNAP-25 in the intoxicated cells is steady but very low, reaching less than 50% after 10 months (28). To determine the duration of action of BoNT/A6 in cultured primary rodent neurons, primary rat spinal cord (RSC) cells, recovery from SNAP-25 cleavage after a pulse of toxin exposure was monitored over 8 months. Up to 6 months, only the BoNT/A cleavage product of SNAP-25 was detected by Western blotting. At 7 months after initial toxin exposure, about 90% of the detected SNAP-25 was cleaved and about 10% uncleaved, indicating beginning of recovery of the cells. After 8 months, about 79% of SNAP-25 remained cleaved (Fig. 5D). Although signs of SNAP-25 recovery were not seen until later compared to previous work on BoNT/A subtypes 1 to 5, the estimated slope of the line when signs of uncleaved SNAP-25 began was similar to that of BoNT/A1, A2, A4, and A5 previously observed, indicating similar persistence of LC activity within cultured neuronal cells (28).

Persistence of BoNT LC activity in human iPSC-derived neurons has previously been shown to be shorter than in primary rat spinal cord neurons, with dose-dependent recovery of the neurons within 3 to 5 months (16, 28). This allowed for estimation of the half-life of activity in human neurons by exposing the neurons to serial dilutions of either BoNT/A1 or BoNT/A6 and determining the EC_50_ at different time points post-toxin exposure. Western blotting and densitometry analyses of triplicate sets of cells with each dilution series harvested at days 3, 39, and 70 post-BoNT exposure resulted in EC_50_ values for BoNT/A6 of ∼0.04 U/50 µl/well (day 3), 0.8 U/50 µl/well (day 39), and 1 U/50 µl/well (day 70) (or 28, 495, and 707 fM, respectively). EC_50_ values of BoNT/A1 were ∼0.42 U/50 µl/well (day 3), 4.0 U/50 µl/well (day 39), and 17 U/50 µl/well (day 70) (or 314, 2,990, and 12,690 fM, respectively) (Fig. 5A and B). The half-life of activity of BoNT/A1 and /A6 in these hiPSC-derived neurons was estimated from the EC_50_ values over time and was similar for both BoNT/A1 and /A6, at approximately 12 days and 14 days, respectively (Fig. 5C). Taken together, these results indicate that the BoNT/A6 LC persists and is enzymatically active in neurons for a similarly long time as BoNT/A1 LCs.

### Onset and duration of action of BoNT/A6 is similar to BoNT/A1 in mice.

**FIG 6:**
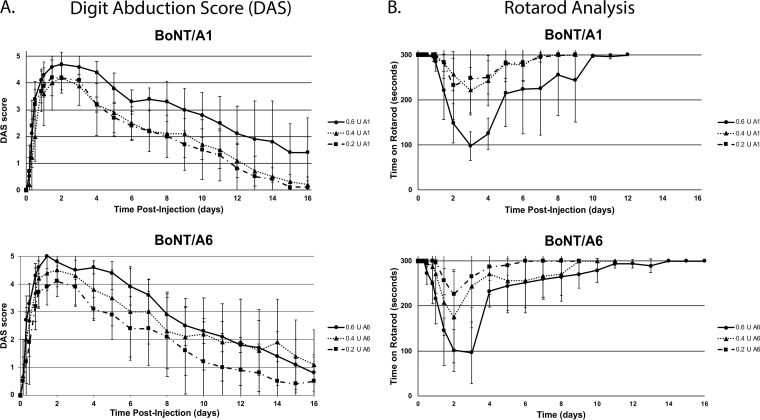
Onset and duration of action of BoNT/A6 and /A1 *in vivo*. Average DAS scores (A) and Rotarod times (B) of mice injected in the right gastrocnemius muscle with 0.6, 0.4, and 0.2 U of BoNT/A1 and BoNT/A6. *n* = 5.

To determine the onset and duration of action of BoNT/A6 compared to BoNT/A1, mice were injected with dilutions of either BoNT ranging from 0.2 to 0.6 U into the right hind gastrocnemius muscle. DAS scores (to measure local paralysis) and Rotarod times (to measure overall motor neuron deficiency) were recorded for each mouse at several time points throughout the first 48 h and then once each day through 16 days postinjection. The injected doses of each toxin were confirmed by IP LD_50_ assay using the same dilutions (data not shown). As previously seen for other BoNTs, the DAS scores and Rotarod recovery times were both dose dependent (Fig. 6). The peak DAS score appeared between 36 and 48 h, and it started to decrease at day 3 to 4, similar to previous reports for BoNT/A2 and slightly earlier than BoNT/A1 (Fig. 6A). By day 16, the DAS score had dropped to ∼1.5 for mice injected with the highest toxin dilution (Fig. 6A), which is similar as previously observed with BoNT/A1, /A2, and /A5 (17). Overall motor neuron deficiency, as measured by Rotarod, was similar for both BoNT/A1 and /A6 (Fig. 6B) and similar to that previously observed for other BoNT/A subtypes (17). These data indicate similar onset and duration of action of BoNT/A6 compared to BoNT/A1 for local paralysis in mice after local intramuscular injection, in addition to similar overall motor neuron deficiency.

### BoNT/A6 remains more localized at the injection site after local intramuscular injection.

BoNT/A2, which enters cultured neuronal cells faster and more efficiently, similar to BoNT/A6, has previously been suggested to remain more localized after intramuscular injection into rodent gastrocnemius muscle than BoNT/A1 (15, 19). To determine if the faster cell entry of BoNT/A6 also leads to less toxin spread away from the injection site after local intramuscular injection, a relative IM LD_50_ (relative to the IP LD_50_ of the same toxin dilutions) in mice was determined for injection of BoNT/A1, /A2, and /A6 into the right gastrocnemius muscle. Toxin dilutions of each BoNT/A subtype were injected either intraperitoneally or intramuscularly in parallel, and mice were scored for survival. Most mice injected with lethal doses IP were found dead the day after injection, with a few dead at 2 days postinjection. However, the mice injected with lethal doses IM died slower, with deaths still occurring over a wide range of time postinjection. Using the Reed and Muench calculation methods, the LD_50_ values were calculated to be 5.6, 4.9, and 5.3 pg for the mice injected IP and 7.9, 9.9, and 10.6 pg for mice injected IM with BoNT/A1, /A2, and /A6, respectively. Thus, the IM LD_50_ for BoNT/A2 and BoNT/A6 both was ∼2 IP LD_50_ units whereas the IM LD_50_ for BoNT/A1 was ∼1.4 IP units (Table 1).

**TABLE 1:** IM LD_50_ of BoNT/A1, /A2, and /A6 (*n* = 10)

BoNT subtype	IP LD_50_ (pg)	IM LD_50_ (pg)	IM LD_50_ (U)
A1	5.6	7.9	1.4
A2	4.9	9.9	2.0
A6	5.3	10.6	2.0

## DISCUSSION

BoNT/A6 is naturally expressed in a dual-toxin-producing strain, CDC41370, isolated from a food-borne botulism case in 1996 (23). In addition to BoNT/A6, this strain also expresses BoNT/B2, complicating the isolation and characterization of the BoNT/A6 toxin. As has been previously accomplished for the isolation of BoNT/FA from a bivalent strain (24), strain CDC41370 was modified to eliminate expression of BoNT/B2. BoNT/A6 was expressed at high levels in the resulting strain CDC41370B2^tox-^, and purification using standard protocols existing for other BoNT/A subtypes resulted in isolation of an ≥95% pure BoNT/A6. As has been previously observed for BoNT/A2, BoNT/A6 had a specific activity similar to BoNT/A1 when injected IP in mice but had about 20-fold higher potency in all tested cell models, including primary rat and mouse spinal cord cultures and hiPSC-derived neurons (Fig. 3). BoNT/A6 had similar *in vitro* LC enzymatic activity as BoNT/A1 in the BoTest but showed significantly faster SNAP-25 cleavage in cultured neurons (Fig. 2 and 4). This earlier onset of intracellular SNAP-25 cleavage combined with similar enzymatic activity of the LC indicates faster cell entry by BoNT/A6 than BoNT/A1 and similar entry to that previously observed for BoNT/A2 (5, 20). These data are consistent with the previous observation that BoNT/A6 may be derived from a genetic recombination event of BoNT/A2 and other BoNT/A subtypes (23) and indicate that there may be common amino acid sequence and structural elements in the HC of BoNT/A6 and /A2 underlying the faster cell entry kinetics.

We have previously observed that BoNT/A3 has a significantly shorter duration of action than BoNT/A1. The duration of action of BoNT/A6 was examined both in cultured neurons and in mice as previously performed for other BoNT/A subtypes (17, 28). Both the cell culture and *in vivo* mouse data indicate similar duration of action of BoNT/A6 and /A1 (Fig. 5 and 6) (17, 28). Interestingly, while persistence of BoNT/A1 and /A6 LC activity in cultured primary rodent neurons was very long, with only about 50% recovery after 10 months (Fig. 5D) (28), persistence of BoNT LC activity in cultured human iPSC-derived neurons was much shorter, with dose-dependent recovery of the neurons within 3 to 5 months, as has previously been shown for other BoNTs (16). This recovery time is similar to the recovery of paralysis of human muscle after local injection with pharmaceutical BoNT/A1. While the reasons for the difference in recovery time between the two cell models are not known, likely explanations include differences in differentiation stages of the cell models, differences in cell composition (>98% pure forebrain-like neurons versus a mixture of spinal cord neurons and glial cells), and the presence of glial cells in the primary neurons versus the pure neuronal population of the hiPSC-derived neurons. Furthermore, the different BoNTs may show different persistence because of different reactivities or mechanisms of BoNT degradation in cells such as ubiquitin-mediated protein degradation. Regardless of the underlying mechanism, this shorter recovery time enabled the estimation of the half-lives of BoNT/A1 and /A6 in human neurons, with the data indicating similar half-lives of ∼12 and ∼14 days for A1 and A6, respectively (Fig. 5C), which is consistent with a similar duration of action in primary rat spinal cord cells and in mice. Together, these data demonstrate a similar duration of action of BoNT/A6 and BoNT/A1, which is consistent with the high degree of amino acid identity of the A6 and A1 LCs, which differ by only one residue (T414A).

Faster cell entry of BoNT/A2 has been suggested to correlate with faster onset of local paralysis after intramuscular injection (15, 17–20). Similar to what was previously observed for BoNT/A2, the onset of local paralysis as measured by DAS appeared slightly earlier for BoNT/A6 than for BoNT/A1 (Fig. 6A), although these observational data rely on qualitative and relatively small differences and will need to be confirmed in larger animals or in a clinical setting. Mice are relatively small animals compared to humans and other animals such as wildlife showing botulism in nature. Therefore, mice and other small animal models require local BoNT doses relatively close to lethal doses for observation of significant local paralysis (0.2 to 0.75 U), with 1 to 2 units being lethal after IM injection (Table 1). Due to the resulting relatively small effective dose range in mice, it may be difficult to quantitatively discern differences in the dose-dependent onset of paralysis time. For botulism in larger animals or in pharmaceutical treatments of humans, smaller relative doses can be injected locally and lead to paralysis, likely due to the larger size of the injected muscle and whole organisms being less prone to systemic distribution of toxin. Therefore, future studies in larger animals or clinical studies will be required to definitively determine whether BoNT/A6 and BoNT/A2 result in a significantly faster onset of pharmaceutical action than BoNT/A1.

The faster and more efficient cell entry and possibly faster onset of action by BoNT/A6 and /A2 than BoNT/A1 raise the questions whether BoNT/A6 and /A2 have the potential to remain more localized within the injection site after *in vivo* intramuscular injection, which would result in fewer side effects during pharmaceutical use due to toxin spread away from the injection site. *In vivo* studies in mice comparing a pharmaceutical preparation of purified BoNT/A2 (with the excipients unknown) to pharmaceutical BoNT/A1 complex indicated a significant difference in the IM LD_50_ for the two toxins (19). Here, we directly compared purified preparations of BoNT/A1, /A2, and /A6 for their lethality in mice after intraperitoneal and intramuscular injection. Importantly, for each subtype the same toxin dilutions and relative concentrations of active BoNT were used for the IM and IP injections, enabling determination of a relative IM LD_50_ dose for each toxin. Based on the calculated LD_50_ determined from a standard MBA with IP injections, IM injections of BoNT/A6 and BoNT/A2 required 2.0 times as much toxin to be lethal as the amount of toxin injected IP, while BoNT/A1 required only 1.4 times as much toxin (Table 1). These data support the previous observation that BoNT/A2 remains more localized after intramuscular injection than BoNT/A1 and indicate that, similar to BoNT/A2, BoNT/A6 also results in less systemic toxin distribution than for BoNT/A1 (19).

Taken together, our results demonstrate several properties of BoNT/A6 that would be beneficial if used as a new BoNT-based pharmaceutical, including faster and more efficient neuronal cell entry than BoNT/A1, similarly long duration of action, and less systemic spread after local injection. Treatments with BoNT/A6 may relieve targeted symptoms faster with fewer risks for side effects and without compromising the long-lasting duration. This study is based on one batch of purified BoNT/A6, and future work with additional batches and pharmacologic preparations of BoNT/A6 will be required to assess safety and effectiveness of BoNT/A6 as a new pharmaceutical. These studies showing different phenotypes in cells and animals for subtypes also provide biological systems for understanding the molecular, cellular, and organismal basis for these different and important properties of BoNTs.

## MATERIALS AND METHODS

### Biosafety, biosecurity, and ethics.

The Johnson laboratory and personnel are registered with the Federal Select Agent Program for research involving botulinum neurotoxins (BoNTs) and BoNT-producing strains of clostridia. The research program, procedures, documentation, security, and facilities are closely monitored by the University of Wisconsin-Madison Biosecurity Task Force, the University of Wisconsin-Madison Office of Biological Safety, the University of Wisconsin-Madison Select Agent Program, and the Centers for Disease Control and Prevention (CDC) as part of the University of Wisconsin-Madison Select Agent Program. All personnel have undergone suitability assessments and completed rigorous and continuing biosafety training, including biosafety level 3 (BSL3) and BSL2 and select agent practices, before participating in laboratory studies involving BoNTs and neurotoxigenic C. botulinum. All animal experiments were approved by and conducted according to guidelines of the University of Wisconsin Animal Care and Use Committee.

### Construction of a BoNT/B2 insertional mutant from the native BoNT/A6-producing C. botulinum strain.

Strain CDC41370 expresses both BoNT/B2 and BoNT/A6 (23). The production of two BoNTs complicates the isolation of pure BoNT/A6. The gene encoding botulinum neurotoxin B2 (GenBank accession number FJ981697) in C. botulinum strain CDC41370 was inactivated with a ClosTron mutagenesis system by insertion of a mobile group II intron between nucleotide 381 and 382 on a sense strand using plasmid pMTL007C-E2::Cbo:bontbvb-381s as previously described for inactivation of the BoNT/B2 gene in C. botulinum strain CDC69016 (15, 24–27, 29). Inactivation of the BoNT/B2 gene was confirmed by PCR, DNA sequencing, and Southern hybridization as previously described using the same primers and probes (24). This resulted in strain CDC41370B2^tox-^, which expressed exclusively BoNT/A6 at high levels. BoNT/A6 (∼150-kDa protein) was then purified from this modified strain, yielding highly pure toxin using the method previously described to produce BoNT/FA (15, 26, 27).

### Botulinum neurotoxins.

BoNT/A1 and /A2 were independently purified (∼150-kDa proteins) from C. botulinum strains Hall A-*hyper* and Kyoto-F as previously described (15, 26, 27). The purity of the toxins was confirmed by spectroscopy and SDS-PAGE. The purified toxins were stored in 0.1 M sodium phosphate buffer, pH 7, with 40% glycerol at −20°C until use. Specific activities of each subtype preparation were determined using an intraperitoneal mouse bioassay (MBA) as previously described (30–33). The specific activities of the BoNTs in mice were 5.6 pg/LD_50_ (A1), 4.9 pg/LD_50_ (A2), and 5.3 pg/LD_50_ (A6). Protein concentrations of purified BoNT/A1, /A2, and /A6 were determined by measuring the absorbance at *A*_278_ and an extinction coefficient of 1.63 and by SDS-PAGE gel analysis.

### BoTest.

The *in vitro* SNARE fragment-leaving BoTest for A/E BoNTs (Biosentinel Pharmaceuticals) was used according to the manufacturer’s instructions. Tenfold serial dilutions of BoNT/A1 and /A6 were prepared in 1× reaction buffer solution containing 5 mM dithiothreitol (DTT) (Sigma) for toxin reduction. The data presented are the average from the results from three independent experiments performed on each subtype, each of which included a control without BoNT present. The plate was incubated at 30°C for 2 h, and emission ratios were determined after addition of reporter to the BoNT dilutions. A BioTek Synergy H1 Hybrid reader recorded the absorbance of each well. The 50% effective concentration (EC_50_) was determined by analyzing the averages and standard deviations for each toxin concentration from the three independent assays using a nonlinear regression (four parameters) in PRISM 6.

### Primary rat (RSC) and mouse spinal cord (MSC) cell assay.

Primary rat and mouse spinal cord cells were prepared as previously described (28, 34). The cells were plated on a 96-well, flat-bottom plate (Techno Plastic Products [TPP]) plate treated with 0.01% poly-l-ornithine (Sigma) and coated with 8.3 µg/cm^2^ growth factor-reduced Matrigel (BD Biosciences). The cells were maintained in culture medium (CM) (Neurobasal medium supplemented with B27, GlutaMAX, and penicillin/streptomycin [Life Technologies]) and allowed to mature for a minimum of 2 weeks. Cells were exposed to BoNT in 50 µl CM per well and incubated at 37°C in a 5% CO_2_ humidified atmosphere for the indicated amount of time. Cells were lysed in 75 µl of 1× LDS lysis buffer and analyzed by Western blotting.

To determine the EC_50_, MSCs and RSCs were exposed to serial (3-fold) dilutions of BoNT in CM. Experiments were performed in at least triplicate, and a no-toxin control was included in each replicate. BoNT dilutions remained on cells for 48 h at 37°C in a 5% CO_2_ humidified atmosphere. BoNT was then removed, cells were lysed in 75 µl of 1× LDS lysis buffer (Life Technologies), and cell lysates were analyzed for the presence of cleaved and uncleaved SNAP-25 by Western blotting as previously described (28, 34).

To determine the duration of action of BoNT/A6 in primary neuronal cells, RSCs were exposed for 72 h to 8 pM BoNT/A6, which is the minimum required to achieve 100% SNAP-25 cleavage in RSC cells. Extracellular BoNT was removed by washing the cells in 0.3 ml of CM three times, and cells were further incubated in culture medium without BoNT as previously described for other BoNT/A subtypes (28). Cells were harvested at 3 days after initial BoNT exposure and monthly thereafter until 8 months postexposure. All time points were tested in quadruplicate and included no-toxin controls. Cleaved and uncleaved SNAP-25 was monitored over time by Western blotting and densitometry.

### Human iPSC-derived neuron cell assay.

iCell GABANeurons (Cellular Dynamics) were stored in liquid nitrogen until use. The cells were plated on a TPP, 96-well, flat-bottom plate treated with 0.01% poly-l-ornithine and 8.3 µg/cm^2^ Matrigel coated. The cells were maintained in iCell Neuron maintenance medium supplemented with iCell Neuron medium supplement (Cellular Dynamics) and matured for about 1 week until toxin exposure.

To determine the EC_50_, the cells were exposed to serial (3-fold) dilutions of BoNT in CM. Experiments were performed in at least triplicate, and a no-toxin control was included in each replicate. BoNT dilutions remained on cells for 48 h at 37°C in a 5% CO_2_ humidified atmosphere. BoNT was then removed, cells were lysed in 50 µl of 1× LDS lysis buffer, and cell lysates were analyzed for the presence of cleaved and uncleaved SNAP-25 by Western blotting and densitometry.

To determine the duration of action of BoNT/A6 in hiPSC-derived neurons, cells were exposed to serial dilutions of BoNT/A6 in culture medium for 72 h, after which time extracellular toxin was removed and cells were washed thoroughly. Cells were returned to the incubator and fed every 2 to 3 days. Cells were harvested on days 3, 39, and 70 postexposure, and cell lysates were analyzed by Western blotting and densitometry.

To compare cell entry kinetics of BoNT/A1, /A2, and /A6, hiPSCs were exposed to equal molarity (67 pM) of each BoNT subtype. Cells were harvested at the indicated time points after toxin addition, and cell lysates were analyzed for SNAP-25 cleavage by Western blotting. Entry kinetics were deduced based on the SNAP-25 cleavage determined by Western blotting analysis and densitometry calculations of at least triplicate samples of each toxin at each time point.

### Western blot analysis.

Western blot analyses were carried out as previously described (28, 34). All cell lysates were separated on 12% Novex NUPAGE gels (Life Technologies) using MES running buffer (Invitrogen) and transferred onto a 0.45-µm PVDF membrane (Millipore). Membranes were incubated in blocking buffer for 30 min and then in anti-SNAP-25 (Synaptic Systems) primary antibody solution overnight. After five 5-min washes with washing buffer (KPL), the membranes were rinsed with ddH_2_O and incubated in secondary anti-mouse antibody (KPL) for one hour. Five additional washes with washing buffer were done, and membranes were rinsed with ddH_2_O and incubated in chemiluminescent substrate (Phosphaglo, KPL) for ∼3 min. Images of bands on membranes were obtained using a Fotodyne FOTO/Analyst FX imager and analyzed by densitometry with TotalLab Quant and PRISM 6 software (GraphPad Software Inc.).

### Onset and duration of action *in vivo*.

Onset and duration of action were determined *in vivo* as previously described (17). Using a 0.3-ml insulin syringe with 5-µl markings, groups of 5 female ICR mice were injected with the indicated concentrations of BoNT/A1 or BoNT/A6 in 10 µl GelPhos (30 mM sodium phosphate, pH 6.3, 0.2% gelatin) into the right gastrocnemius muscle. Local paralysis was measured by the digit abduction score (DAS) for the right hind limb of each mouse at several time points within the first 48 h after injection and every 24 h thereafter on a 0 to 5 scale (17). At the same time points, Rotarod analysis (MED-Associates) was performed on each mouse (after DAS determination). Each mouse attempted to run for a total of 5 min while the Rotarod increased in speed from 4 to 40 rpm. Rotarod analysis was ceased for a group once all mice ran the full 5 min 2 times consecutively.

### Determination of intramuscular LD_50_ in mice.

Groups of 10 female ICR mice (Harlan) were injected with the indicated amounts of BoNT/A1, /A2, or /A6 in 10 µl GelPhos buffer using a 0.3-ml insulin syringe (BD) into the right gastrocnemius muscle. In parallel, groups of 5 mice received intraperitoneal injections of the same BoNT dilutions in 0.5 ml GelPhos buffer using a 0.5-ml insulin syringe (BD). Mice were observed through 6 days postinjection, and any deaths were recorded. The LD_50_ in pg was calculated for both the intramuscularly (IM) and intraperitoneally (IP) injected mice using the Reed and Muench method (35). Based on the IP data and a unit definition of 1 unit = the amount of BoNT required to result in death of 50% of mice after IP injection within 4 days, the specific activity of each BoNT was determined in units (IP LD_50_) for the toxin dilutions used in this comparative assay. The IM LD_50_ in units was calculated based on the IP LD_50_ values.
