# Supplementary material for: Isolation and Characterization of the Novel Botulinum Neurotoxin A Subtype 6
Source: mSphere. 2018 Oct 24;3(5):e00466-18. doi: 10.1128/mSphere.00466-18 (PMC6200982; doi:10.1128/mSphere.00466-18)
Supplement: FIG S1 [file sph005182674sf1.pdf]

```
A2      MPFVNKQFNYKDPVNGVDIAYIKIPNAGQMQPVKAFKIHNKIWVIPERDTFTNPEEGDLN 60
A1      MPFVNKQFNYKDPVNGVDIAYIKIPNAGQMQPVKAFKIHNKIWVIPERDTFTNPEEGDLN 60
A6      MPFVNKQFNYKDPVNGVDIAYIKIPNAGQMQPVKAFKIHNKIWVIPERDTFTNPEEGDLN 60
        ************************************************************

A2      PPPEAKQVPVSYYDSTYLSTDNEKDNYLKGVTKLFERIYSTDLGRMLLTSIVRGIPFWGG 120
A1      PPPEAKQVPVSYYDSTYLSTDNEKDNYLKGVTKLFERIYSTDLGRMLLTSIVRGIPFWGG 120
A6      PPPEAKQVPVSYYDSTYLSTDNEKDNYLKGVTKLFERIYSTDLGRMLLTSIVRGIPFWGG 120
        ************************************************************

A2      STIDTELKVIDTNCINVIQPDGSYRSEELNLVIIGPSADIIQFECKSFGHDVLNLTRNGY 180
A1      STIDTELKVIDTNCINVIQPDGSYRSEELNLVIIGPSADIIQFECKSFGHEVLNLTRNGY 180
A6      STIDTELKVIDTNCINVIQPDGSYRSEELNLVIIGPSADIIQFECKSFGHEVLNLTRNGY 180
        **************************************************:*********

A2      GSTQYIRFSPDFTFGFEESLEVDTNPLLGAGKFATDPAVTLAHELIHAEHRLYGIAINPN 240
A1      GSTQYIRFSPDFTFGFEESLEVDTNPLLGAGKFATDPAVTLAHELIHAGHRLYGIAINPN 240
A6      GSTQYIRFSPDFTFGFEESLEVDTNPLLGAGKFATDPAVTLAHELIHAGHRLYGIAINPN 240
        ************************************************ ***********

A2      RVFKVNTNAYYEMSGLEVSFEELRTFGGHDAKFIDSLQENEFRLYYYNKFKDVASTLNKA 300
A1      RVFKVNTNAYYEMSGLEVSFEELRTFGGHDAKFIDSLQENEFRLYYYNKFKDIASTLNKA 300
A6      RVFKVNTNAYYEMSGLEVSFEELRTFGGHDAKFIDSLQENEFRLYYYNKFKDIASTLNKA 300
        ****************************************************:*******

A2      KSIIGTTASLQYMKNVFKEKYLLSEDTSGKFSVDKLKFDKLYKMLTEIYTEDNFVNFFKV 360
A1      KSIVGTTASLQYMKNVFKEKYLLSEDTSGKFSVDKLKFDKLYKMLTEIYTEDNFVKFFKV 360
A6      KSIVGTTASLQYMKNVFKEKYLLSEDTSGKFSVDKLKFDKLYKMLTEIYTEDNFVKFFKV 360
        ***:***************************************************:****

A2      INRKTYLNFDKAVFRINIVPDENYTIKDGFNLKGANLSTNFNGQNTEINSRNFTRLKNFT 420
A1      LNRKTYLNFDKAVFKINIVPKVNYTIYDGFNLRNTNLAANFNGQNTEINNMNFTKLKNFT 420
A6      LNRKTYLNFDKAVFKINIVPKVNYTIYDGFNLRNTNLAANFNGQNTEINNMNFAKLKNFT 420
        :*************:*****. **** *****:.:**::**********. **::*****

A2      GLFEFYKLLCVRGIIPFKTKSLDEGYNKALNDLCIKVNNWDLFFSPSEDNFTNDLDKVEE 480
A1      GLFEFYKLLCVRGIITSKTKSLDKGYNKALNDLCIKVNNWDLFFSPSEDNFTNDLNKGEE 480
A6      GLFEFYKLLCVRGIITSKTKSLDKGYNKALNDLCIKVNNWDLFFSPSEDNFTNDLNKGEE 480
        ***************  ******:*******************************:* **

A2      ITADTNIEAAEENISLDLIQQYYLTFDFDNEPENISIENLSSDIIGQLEPMPNIERFPNG 540
A1      ITSDTNIEAAEENISLDLIQQYYLTFNFDNEPENISIENLSSDIIGQLELMPNIERFPNG 540
A6      ITSDTNIEAAEENISLDLIQQYYLTFNFDNEPENISIENLSSDIIGQLELMPNIERFPNG 540
        **:************************:********************* **********

A2      KKYELDKYTMFHYLRAQEFEHGDSRIILTNSAEEALLKPNVAYTFFSSKYVKKINKAVEA 600
A1      KKYELDKYTMFHYLRAQEFEHGKSRIALTNSVNEALLNPSRVYTFFSSDYVKKVNKATEA 600
A6      KKYELDKYTMFHYLSAQEFEHGKSRIDLTNSVNEALLNPSHVYTFFSSDYVKKVNKATEA 600
        ************** *******.*** ****.:****:*. .******.****:***.**

A2      FMFLNWAEELVYDFTDETNEVTTMDKIADITIIVPYIGPALNIGNMLSKGEFVEAIIFTG 660
A1      AMFLGWVEQLVYDFTDETSEVSTTDKIADITIIIPYIGPALNIGNMLYKDDFVGALIFSG 660
A6      AMFLGWVEQLVYDFTDETSEVSTTDKIADITIIIPYIGPALNIGNMLYKDDFVGALIFSG 660
         ***.*.*:*********.**:* *********:************* *.:** *:**:*

A2      VVAMLEFIPEYALPVFGTFAIVSYIANKVLTVQTINNALSKRNEKWDEVYKYTVTNWLAK 720
A1      AVILLEFIPEIAIPVLGTFALVSYIANKVLTVQTIDNALSKRNEKWDEVYKYIVTNWLAK 720
```

```
A6        AVILLEFIPEIAIPVLGTFAIVSYIANKVLTVQTINNALSKRNEKWDEVYKYTVTNWLAK 720
          .* :****** *:**:****:**************:**************** *******

A2        VNTQIDLIREKMKKALENQAEATKAIINYQYNQYTEEEKNNINFNIDDLSSKLNESINSA 780
A1        VNTQIDLIRKKMKEALENQAEATKAIINYQYNQYTEEEKNNINFNIDDLSSKLNESINKA 780
A6        VNTQIDLIREKMKKALENQAEATKAIINYQYNQYTEEEKNNINFNIDDLSSKLNESINSA 780
          *********:***:********************************************.*

A2        MININKFLDQCSVSYLMNSMIPYAVKRLKDFDASVRDVLLKYIYDNRGTLVLQVDRLKDE 840
A1        MININKFLNQCSVSYLMNSMIPYGVKRLEDFDASLKDALLKYIYDNRGTLIGQVDRLKDK 840
A6        MININKFLDQCSVSYLMNSMIPYAVKRLKDFDASVRDVLLKYIYDNRGTLIGQVDRLKDK 840
          ********:**************.****:*****::*.************: *******:

A2        VNNTLSADIPFQLSKYVDNKKLLSTFTEYIKNIVNTSILSIVYKKDDLIDLSRYGAKINI 900
A1        VNNTLSTDIPFQLSKYVDNQRLLSTFTEYIKNIINTSILNLRYESNHLIDLSRYASKINI 900
A6        VNNTLSTDIPFQLSKYVDNQRLLSTFTEYIKNIINTSILSLRYENNHLIDLSRYASKINI 900
          ******:************::************:*****.: *:.:.*******.:****

A2        GDRVYYDSIDKNQIKLINLESSTIEVILKNAIVYNSMYENFSTSFWIKIPKYFSKINLNN 960
A1        GSKVNFDPIDKNQIQLFNLESSKIEVILKNAIVYNSMYENFSTSFWIRIPKYFNSISLNN 960
A6        GSRVNFDPIDKNQIQLFNLESSKIEVILKNAIVYNSMYENFSTSFWIKIPKYFSEISLNN 960
          *.:* :* ******:*:*****.************************:*****..*.***

A2        EYTIINCIENNSGWKVSLNYGEIIWTLQDNKQNIQRVVFKYSQMVNISDYINRWIFVTIT 1020
A1        EYTIINCMENNSGWKVSLNYGEIIWTLQDTQEIKQRVVFKYSQMINISDYINRWIFVTIT 1020
A6        EYTIINCIENNSGWKVSLNYGEIIWTLQDNKQNIQRVVFKYSQMVAISDYINRWIFITIT 1020
          *******:*********************.::  **********: **********:***

A2        NNRLTKSKIYINGRLIDQKPISNLGNIHASNKIMFKLDGCRDPRRYIMIKYFNLFDKELN 1080
A1        NNRLNNSKIYINGRLIDQKPISNLGNIHASNNIMFKLDGCRDTHRYIWIKYFNLFDKELN 1080
A6        NNRLTKSKIYINGRLIDQKPISNLGNIHASNKIMFKLDGCRDPRRYIMIKYFNLFDKELN 1080
          ****.:*************************:********** :*** ************

A2        EKEIKDLYDSQSNSGILKDFWGNYLQYDKPYYMLNLFDPNKYVDVNNIGIRGYMYLKGPR 1140
A1        EKEIKDLYDNQSNSGILKDFWGDYLQYDKPYYMLNLYDPNKYVDVNNVGIRGYMYLKGPR 1140
A6        EKEIKDLYDSQSNSGILKDFWGNYLQYDKPYYMLNLFDPNKYVDVNNVGIRGYMYLKGSR 1140
          *********.************:*************:**********:********** *

A2        GSVVTTNIYLNSTLYEGTKFIIKKYASGNEDNIVRNNDRVYINVVVKNKEYRLATNASQA 1200
A1        GSVMTTNIYLNSSLYRGTKFIIKKYASGNKDNIVRNNDRVYINVVVKNKEYRLATNASQA 1200
A6        STLLTTNIYLNSGLYMGTKFIIKKYASGNKDNIVRNNDRVYINVVVNNKEYRLATNASQA 1200
          .:::******** ** *************:****************:*************

A2        GVEKILSALEIPDVGNLSQVVVMKSKDDQGIRNKCKMNLQDNNGNDIGFIGFHLYDNIAK 1260
A1        GVEKILSALEIPDVGNLSQVVVMKSKNDQGITNKCKMNLQDNNGNDIGFIGFHQFNNIAK 1260
A6        GVEKILSALEIPDIGNLSQVVVMKSKNDQGIRNKCKMNLQDNNGNDIGFIGFHKFNDIYK 1260
          *************:************:**** ********************* :::* *

A2        LVASNWYNRQVGKASRTFGCSWEFIPVDDGWGESSL  1296
A1        LVASNWYNRQIERSSRTLGCSWEFIPVDDGWGERPL  1296
A6        LVASNWYNRQIEISSRTFGCSWEFIPVDDGWGEKPL  1296
          **********:  :***:***************  *
```
