# Supplementary figures and images for: Isolation and Characterization of the Novel Botulinum Neurotoxin A Subtype 6
Source: mSphere. 2018 Oct 24;3(5):e00466-18. doi: 10.1128/mSphere.00466-18 (PMC6200982; doi:10.1128/mSphere.00466-18)

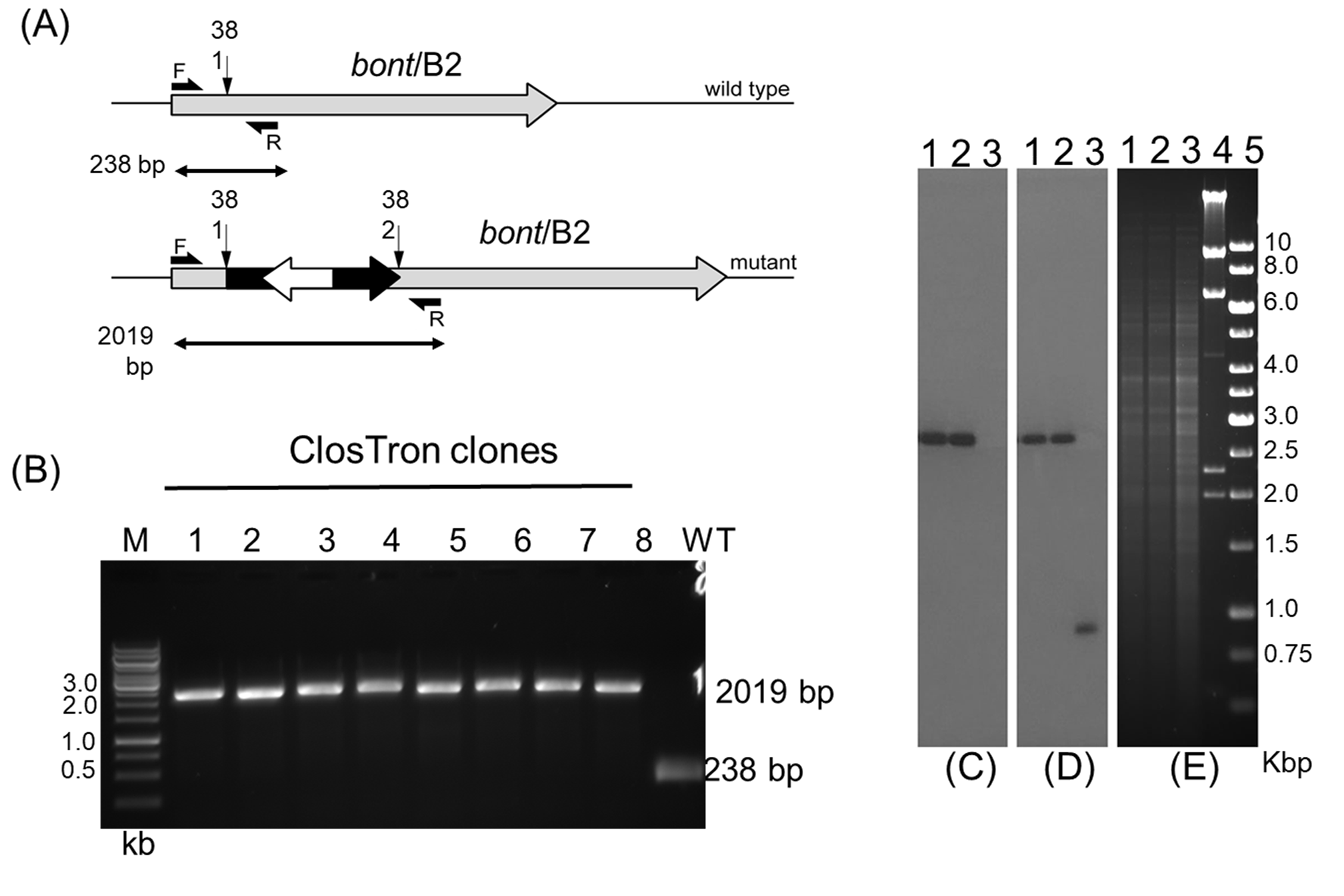

Supplement: FIG S2 [file sph005182674sf2.tif]

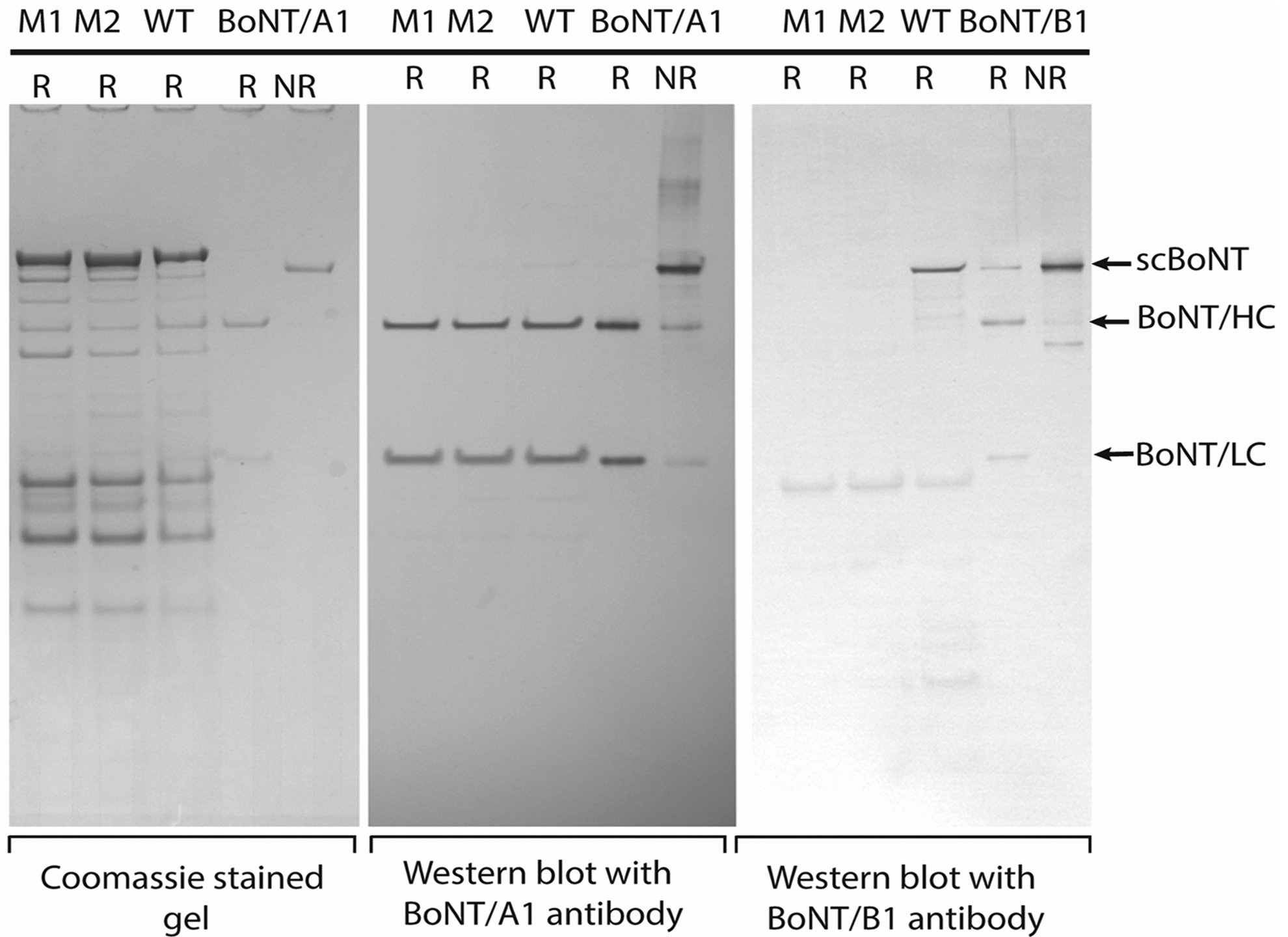

Supplement: FIG S3 [file sph005182674sf3.tif]

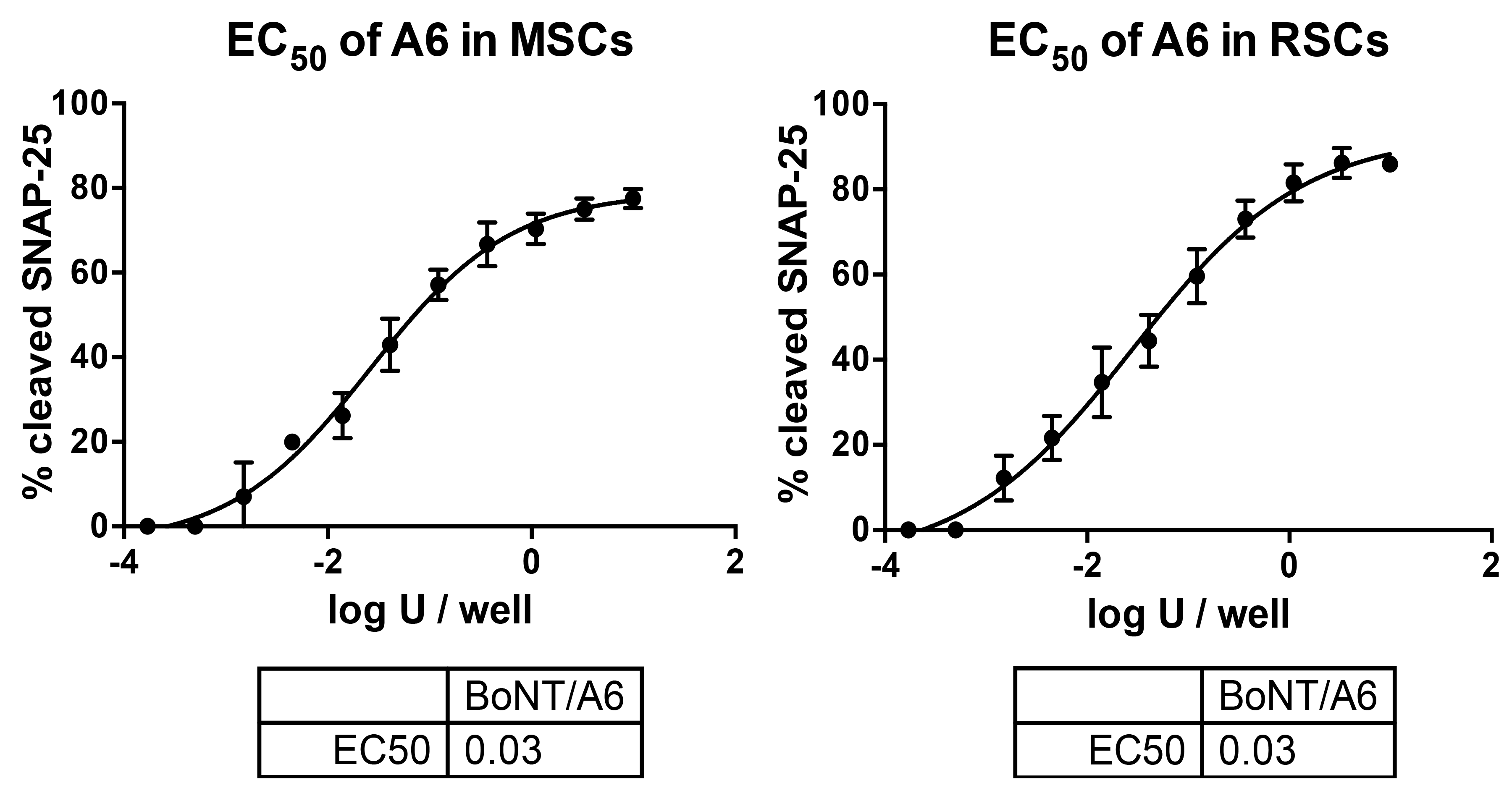

Supplement: FIG S4 [file sph005182674sf4.tif]
